# *Lactobacillus johnsonii* is a dominant *Lactobacillus* in the murine oral mucosa and has chitinase activity that compromises fungal cell wall integrity

**DOI:** 10.1128/mbio.02416-24

**Published:** 2024-09-17

**Authors:** Roberto Vazquez-Munoz, Angela Thompson, Takanori Sobue, Anna Dongari-Bagtzoglou

**Affiliations:** 1 Department of General Dentistry, the University of Connecticut Health Center, Farmington, Connecticut, USA; Geisel School of Medicine at Dartmouth, Hanover, New Hampshire, USA

**Keywords:** *Candida albicans*, oropharyngeal candidiasis, *Lactobacillus johnsonii*, oral microbiome, probiotic therapy, chitinase activity

## Abstract

The oral microbiome is a critical determinant of health and disease, as interactions between oral microorganisms can influence their physiology and the development or severity of oral infections. Lactobacilli have a widely recognized antagonistic relationship with *Candida albicans* and may exhibit probiotic properties that limit oral fungal infection. We previously reported that *Lactobacillus johnsonii* strain MT4, an oral strain isolated from C57BL/6 mice, can induce global changes in the murine oral microbiome and has anti-*Candida* activity *in vitro*. To build on this information, we analyzed its abundance on the mouse oral mucosa, tested its impact on the severity and progression of oropharyngeal candidiasis (OPC) in a mouse model, and further explored the mechanism of antifungal activity *in vitro*. Our findings reveal that *L. johnsonii* MT4 is a dominant cultivable *Lactobacillus* in the oral mucosa of C57BL/6 mice. Strain MT4 has chitinase activity against *C. albicans*, which damages the cell wall and compromises fungal metabolic activity. Oral inoculation with strain MT4 causes a reduction in the *Candida*-induced rise in the abundance of oral enterococci and oral mucosal damage. This research underscores the potential of *L. johnsonii* strain MT4 as a novel probiotic agent in the prevention or management of OPC, and it contributes to a better understanding of the role of oral bacterial microbiota role in the pathogenesis of fungal infections.

**IMPORTANCE:**

The interactions between the opportunistic pathogen *Candida albicans* and resident oral bacteria are particularly crucial in maintaining oral health. Emerging antifungal drug-resistant strains, slow-paced drug discovery, and the risk of side effects can compromise the effectiveness of current treatments available for oropharyngeal candidiasis. This study advances the search for alternative microbiome-targeted therapies in oral fungal infections. We report that *Lactobacillus johnsonii* strain MT4 prevents the *Candida*-induced bloom of dysbiotic oral enterococci and reduces oral mucosal lesions in an oropharyngeal candidiasis murine model. We also show that this strain directly compromises the cell wall and reduces fungal metabolic activity, partly due to its chitinase activity.

## INTRODUCTION

Oropharyngeal candidiasis (OPC) is the most prevalent oral mycosis, primarily afflicting individuals with compromised immune systems and, under certain conditions, impacting those with intact immunity (1–5). *Candida albicans* is the primary etiological agent in OPC (6, 7). This fungal opportunistic pathogen can induce oral mucosal bacterial dysbiosis, characterized by reduced microbiome diversity and the expansion of enterococcal and streptococcal species, which may potentiate fungal virulence and exacerbate the infection (8–12).

The conventional therapeutic approach for OPC involves antifungal agents; however, the emergence of multidrug-resistant *Candida* strains and the limited development of new antifungal drugs pose significant challenges to effective treatment (13). Novel strategies, including microbiome-based therapeutics, are being developed to address these challenges. There is a growing interest in using beneficial bacteria (probiotics) (14) with antimicrobial activity. In particular, certain *Lactobacillus* species display antifungal activity against *C. albicans* (15), can mitigate the severity of candidiasis in murine gut infection models (16), and modulate the mucosal bacterial community composition (17, 18).

In a previous study, we established that a sucrose-enriched diet augmented the oral abundance of *Lactobacillus johnsonii* in *Candida*-infected mice, which was associated with a reduction in fungal virulence in a cortisone-immunosuppressed murine oral infection model (19). We isolated the novel *L. johnsonii* strain MT4 from the oral cavity of these mice, sequenced its genome (20), and characterized its phenotype *in vitro* (21). Strain MT4 exhibited significant anticandida activity in both planktonic and biofilm growth models *in vitro*, and we identified genetic and phenotypic traits potentially associated with this activity (21).

In this follow-up study, we explored further the mechanisms of antifungal activity of *L. johnsonii* strain MT4 and assessed its role in controlling oral candidiasis in a mouse model. We found that strain MT4 inhibits fungal metabolic activity, has chitinase-like properties, and damages the fungal cell wall *in vitro*. In a mouse OPC model, the MT4 strain attenuates fungal virulence by reducing mucosal histopathology. Also, strain MT4 significantly reduced the overgrowth of resident opportunistic enterococci during oral infection. Our study highlights the importance of exploring probiotic strategies that leverage the interactions between resident host bacterial microbiota and opportunistic pathogenic fungi, providing a foundational step toward innovative treatments beyond traditional antifungal medications.

## MATERIALS AND METHODS

### Study animals

Female C57BL/6 mice, 3- to 5-week-old, from Jackson Laboratories, were utilized in all experiments. Sex is not a significant biological risk factor in oropharyngeal candidiasis (22, 23). Before interventions, mice were acclimatized for 1–2 weeks. Mice were randomly assigned to experimental groups one day before the experiments, housed in corresponding cages, tagged, and weighed. All animal studies in this work adhered to the Guide for the Care and Use of Laboratory Animals of the National Institutes of Health, the Animal Welfare Act (AWA) federal regulations, and the University of Connecticut Institutional Animal Use and Care Committee (IACUC) guidelines. All protocols employed in this study received approval from the UConn Health IACUC committee (IACUC protocol #AP2008-1225).

### Characterization of oral lactobacilli in C57BL/6 mice

Within 12 days of their arrival at the animal care facility, swabs were taken from the oral cavity of mice (*n* = 63) from four different shipment batches. Cotton swabs were vortexed in 100 µL of sterile PBS, cultured on Rogosa (Himedia) agar plates, and incubated anaerobically at 37°C for 48–72 h. Bacterial colonies were selected based on their morphology (Fig. S1A). *Lactobacillus*-like and non-*Lactobacillus*-like colonies on Rogosa agar were randomly selected (5–10 colonies/mouse) and independently transferred to 500 µL Eppendorf tubes containing 50 µL of DNase-free water and stored at −80°C. Cells were disrupted with two cycles of heat (95°C for 5 min) and freezing (−80°C, 3 h). DNA in cell fragments from 347 *Lactobacillus*-like colonies and 42 non-*Lactobacillus*-like colonies was PCR-amplified using 16S rRNA gene primers for *Lactobacillus* at the genus level (24), and the *L. johnsonii* strain-specific primers (Table S1). Strain-specific primers were designed using the Primer-BLAST tool (NCBI, https://www.ncbi.nlm.nih.gov/tools/primer-blast/) by targeting a gene encoding for the hypothetical protein ONDMJEFH_01749. A comparative genomic analysis performed with Roary v3.13.0 (25) on 17 *L*. *johnsonii* strains phylogenetically closest to strain MT4 (21) showed that this single copy gene is found only in strains MT4 and NCK2677, a fecal isolate from C57/BL6 mice (26) which shares a >99.9% average nucleotide identity with MT4 (21). Primer specificity was additionally confirmed *in silico* by blasting the primers against bacterial, murine, and fungal DNA sequences in the NCBI RefSeq database. Primers were synthesized by Integrated DNA Technologies, Inc., and their specificity was confirmed experimentally against murine (C57BL/6 mice), fungal (*C. albicans* SC5314), and bacterial DNA from ATCC reference strains and murine isolates. These included the murine oral *Enterococcus faecalis* strain #13 (8) and ATCC 29212, *Streptococcus oralis* strain 34, *Lactobacillus johnsonii* strain ATCC33200, *L. paracasei* strain ATCC334, and a murine *L. murinus* isolate. Primers and amplification conditions are described in Table S1. The size of amplicon products was evaluated *via* Agarose Gel Electrophoresis (AGE; 2% agarose gel, 100 mV, for 90 min) (Fig. S1B).

### Microbial strains and culture conditions

*C. albicans* strain SC5314 was subcultured overnight in Yeast-Peptone-Dextrose (YPD) broth (Sigma, USA) aerobically at 30°C on a shaker before all experiments. In some experiments, we used *C. albicans* strains DAY286 and FJS5 (a DAY286-derived *cht2/cht2* mutant with reduced chitinase activity [described in reference (27)], kindly provided by Prof. Aaron Mitchell and strains SN250 and SN670 [an SN250-derived *cht2/cht2* mutant (28)], kindly provided by Prof. Clarissa Nobile. *Lactobacillus johnsonii* strain MT4 was subcultured overnight in Man, Rogosa, and Sharpe (MRS) broth (USBiological, USA) anaerobically at 37°C for 24 h. Overnight bacterial cultures were washed with PBS, adjusted *via* spectrophotometry to OD_600_ = 0.2, and grown to a final OD_600_ = 0.7–1 anaerobically prior to experiments.

### Effect of MT4 on fungal metabolic activity

*L. johnsonii* strain MT4 inhibits the growth of 24–48 h *C. albicans* biofilms *in vitro* (21); we thus determined whether this is due to a reduction in fungal metabolic activity using a 4-h coculture system. *C. albicans* (10^5 cells/mL) was cocultured with MT4 (10^6^–10^7^ bacterial cells/mL) in Brain Heart Infusion (BHI) broth (Difco), aerobically in 5% CO_2_ at 37°C. MT4 was either mixed with *C. albicans* (direct contact) or separated by placing the bacteria in 0.4-µM pore-size transwell insert filters (Costar, USA). To assess fungal metabolic activity, organisms were stained with the fluorescent stain FUN-1 (Invitrogen, USA) (5 µM) for 90 min at 30°C and counterstained with Calcofluor White (CW; Sigma, USA) (25 µM) for 10 min at room temperature (RT), protected from light. Monocultures and heat-killed *C. albicans* were used as controls. The effect of MT4 on fungi was assessed *via* fluorescent microscopy. Images were obtained using a Zeiss Axio Imager M1 epifluorescence microscope with a 63× objective magnification and the fluorescein isothiocyanate (FITC) and Cy3 filters set with excitation at 480 nm and emission ≥530 nm and ≥620, respectively. Images were post-processed in the Zen v3.3 Blue edition software (Zeiss).

### Bioinformatics analysis of a putative MT4 chitinase

Prior genomic analysis of strain MT4 suggested the presence of a putative peptidoglycan hydrolase with chitinase activity (21). To compare the MT4 putative chitinase with Msp1/p75, a bacterial peptidoglycan hydrolase with chitinase properties, protein sequences of Msp1/p75, and other chitinases were searched in the UniProt database (https://www.uniprot.org/uniprotkb) and blasted [TBLASTN v2.16.0+, https://blast.ncbi.nlm.nih.gov/ (29)] against *L. johnsonii* strain MT4 whole-genome sequence [WGA project: JAJQJG (20)]. The putative peptidoglycan hydrolase (GenBank: MCF1582664.1) from MT4 was aligned with Msp1/p75 sequences in UniProt (Clustal Omega v1.2.4) to assess their homology percentage using the percent identity matrix. To assess potential structural similarities between these products, the 3D structure of the MT4 putative chitinase and MSp1/p75 sequences was modeled in the Swiss model (https://swissmodel.expasy.org/) and corroborated in AlphaFold [https://alphafold.ebi.ac.uk/ (30)]. Next, the 3D structure from strain MT4 putative chitinase was superposed to the structures of Msp1/p75 (UniProt A0AA43SZT2, A0A7D9N840, and A0A9 × 4XB52) in the Swiss model. The chitin-binding NlpC/P60 domain (SSTAQTVVSAAQSQIGKPYVWGATGPNAYDCSGLVQYAYSQAGKNVGRTTYQQAGAGQHISVSQAQAGDILMWGDYHDAIYVGNNQYVHAPQPGQNVTQASISSYFMPDYAIRVN) was identified as a shared sequence and was included in the alignment analysis and the 3D modeling.

### Chitinase activity assay

Chitinase activity (EC 3.2.1.14) in strain MT4 was assayed based on chitin-azure breakdown, a chromogenic substrate (Sigma). Overnight cultures of strain MT4 were adjusted to OD_600_ = 0.2 *via* spectrophotometry, subcultured to OD_600_ = 0.7 (mid-log phase), and then incubated anaerobically overnight in MRS broth without dextrose containing 0.3% m/v chitin-azure as a substrate. Chitinase (from *Streptomyces griseus,* 0.3%, Sigma) was used as a positive control for chitinase activity, and Bisdionin C (1 mM, Sigma), a chitinase inhibitor (31), as a negative control. After incubation, samples were boiled at 100°C for 5 min, centrifuged, transferred in triplicates into a 96-well plate, and incubated for 30 min at RT. Chitinase activity was assessed by measuring the absorbance of the supernatants (λ = 560 nm) in a Synergy 2 plate Reader using the Gen5 software (BioTek). Results were expressed as the fold change of absorbance with MT4 over the chitin-azure-supplemented media alone (i.e., no MT4).

To test whether the chitinase activity contributes to strain MT4 anticandida activity, *C. albicans* (10^5^ yeast cells/mL) was cocultured with bacteria in a 1:1 and 1:10 yeast-lactobacilli cell ratio, with and without Bisdionin C (1 mM), in Brain-Hearth infusion (BHI) broth, aerobically with 5% CO_2_ at 37°C for 4 h. Single-species cultures were used as controls. Fungal metabolic activity was assessed using the colorimetric tetrazolium salt (XTT) assay. Absorbance was measured in an Opsys MR (thermos Labsystems) plate reader and converted to the percentage of viability using the fungal single-species culture absorbance as the 100% viability value and subtracting the corresponding MT4 monoculture absorbance from the coculture absorbance values. BHI was used in these experiments to maintain a near-neutral pH due to its superior buffering capacity compared to MRS (21).

To test whether chitinase activity from *L. johnsonii* may produce chito-oligomers on fungal cell walls, we used wheat germ agglutinin (WGA), a plant protein that binds specifically to internal N-acetylated sugar residues exposed by chitinases (32). Paraformaldehyde (4% PFA)-fixed *C. albicans* was incubated with strain MT4 planktonically overnight in MRS without dextrose, at 1:10 fungal:bacterial cell ratio. Heat-killed MT4 and live MT4 plus Bisdionin C (1 mM) were used as negative controls. Fungal cells were stained with 0.5% WGA-Alexa Flour 633 fluorescent conjugate (Invitrogen) at 37°C for 30 min, followed by Calcofluor White (25 µM) for 5 min at RT, protected from light. Samples were analyzed *via* fluorescence-activated cell sorting (FACS) in a BD FACSymphony A5 SE Cell Analyzer flow cytometer with the BD FACSDiva software. 20,000 *Candida* cells per experimental condition were analyzed in four independent experiments to obtain the mean fluorescence intensity (MFI). Results were expressed as the fold MFI of MT4-treated *Candida* cells over untreated *Candida* cells. Fluorescent microscopy images from these cultures were also obtained to examine the staining pattern on the fungal cell wall using a Zeiss Axio Imager M1 epifluorescence microscope with a 63× objective magnification and the AlexaFluor 633 (λ_ex_=631, λ_em_=650) and Calcofluor White (λ_ex_=350, λ_em_=432) filters. Images were post-processed in the Zen v3.3 Blue edition software (Zeiss).

### Effect of *L. johnsonii* MT4 on *C. albicans* cell wall

The impact of MT4 on the ultrastructure of the fungal cell wall was analyzed *via* transmission electron microscopy (TEM). Paraformaldehyde-fixed *C. albicans* cells were incubated with MT4 overnight in MRS without dextrose at a 1:10 fungal:bacterial cell ratio, as above. Untreated and chitinase-treated cells were used as negative and positive controls, respectively. Samples were fixed in 2.5% glutaraldehyde and post-fixed with 1% OsO_4_ + 0.8% Ferricyanide in 0.1M cacodylate buffer (1 h, RT) and stained with 1% uranyl acetate (1 h, RT). EtOH-dehydrated samples were embedded into Spurr’s epoxy resin with a series of Resin-Poly/Bed812 (Polysciences Inc. USA) dilutions and then incubated at 60°C for 48 h for embedding. 70 nm sections obtained with a diamond knife in a Laica ultramicrotome (EM UC7) were mounted in 3 mm copper grids. Micrographs were acquired in a Hitachi H-7650 TEM (80 kV, 8,000×–15,000×). Ten random cells per treatment were selected for assessing changes in the cell wall by measuring the total, inner, and outer cell wall thickness in four different sites per cell using ImageJ (NIH) and averaged. Data were analyzed and graphed in GraphPad Prism 10.

### *C. albicans-L. johnsonii* murine co*-*infection model

**Fig 1:**
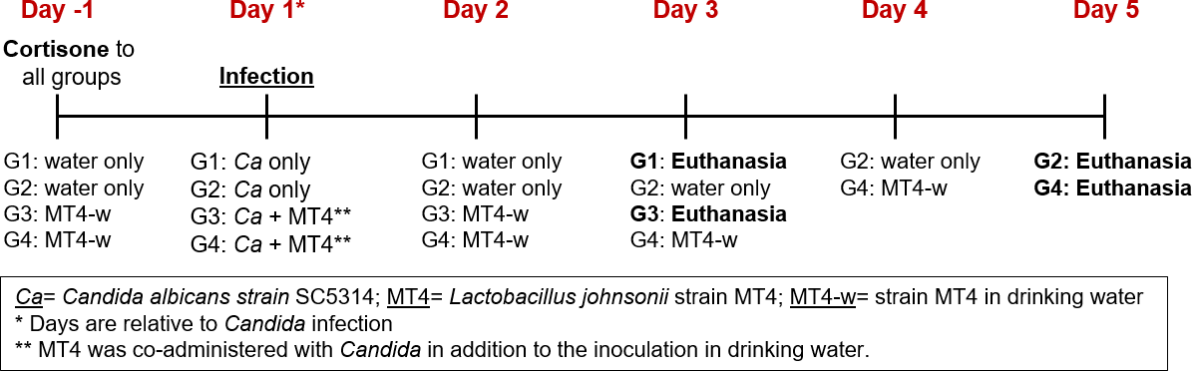
Experimental groups and timeline for *Candida* oral infection, with and without strain MT4 inoculation.

Mice were fed with a powdered diet (Teklad 2918, Envigo) for the duration of the experiment. This diet requires lower levels of immunosuppression (a single cortisone injection), promotes the development of oral lesions within 48 h, and mice begin to recover from infection by day 5 (33). One day before *C. albicans* infection, mice were immunosuppressed with cortisone (225 mg/kg body weight, injected subcutaneously). The following day, mice were anesthetized with ketamine:xylazine (90–100 and 10 mg/kg of body weight, respectively, *via* intramuscular injection) and infected subglossally with *C. albicans* (6 × 10^8^ yeast cells in cotton pellets). Cotton pellets with PBS only were administered orally under anesthesia to the control groups. To test the effect of *L. johnsonii* strain MT4 on the severity and progression of OPC, MT4 (10^9^ cells) was orally co-administered with *Candida* on the day of infection in some *Candida*-infected groups. *L. johnsonii* strain MT4 was also provided daily (10^9^ cells/mL, in drinking water) *ad libitum* for the duration of the experiment. Groups were euthanized either on day 3 or day 5 post-*Candida* infection to test whether MT4 can ameliorate infection or expedite recovery from OPC (Fig. 1).

### Data/sample collection and analyses

#### Body weight

Changes in body weight were monitored every other day as a sign of animal morbidity and expressed as a percentage of initial weight. Food consumption (grams) and water (mL) were measured daily to assess infection-related distress.

#### Assessment of tongue lesions

Tongues were removed aseptically at necropsy, photographed, and images were saved as jpg files. Images were subsequently analyzed using the NIH Image J software (http://rsb.info.nih.gov/ij), and data were expressed as percent surface area covered by biofilm (total surface area of white lesions/entire tongue dorsal surface area). Previous studies have shown that this assessment method correlates well with histologic evidence of tissue damage (10, 34).

#### Histopathological analysis

Excised tongues were fixed with 4% paraformaldehyde at 4°C, processed for paraffin embedding, and sectioned for histopathological analyses. Sections were deparaffinized and stained with FITC-conjugated anticandida antibody (Meridian Life Science, #B65411F) and anti-E-cadherin (BD Transduction, #610182), as primary antibodies. The integrity of the mucosal barrier was assessed using a secondary antibody for E-cadherin conjugated with Alexa Fluor 568 (Invitrogen, #A11031) (8), followed by Hoechst 33258 (Invitrogen, H3569) for counterstaining (35). Tissue sections were also stained with hematoxylin-eosin (H&E) to assess changes in the structural integrity. Images were obtained using a Zeiss Axio Imager M1 microscope, processed in Zen v3.3 Blue edition (Zeiss), and stitched in Imaris Stitcher v10.0.0 (Oxford Instruments). The final images were saved in tiff format.

#### CFU quantification

Excised tongues were weighed and homogenized with a tissue homogenizer (Polytron PT1200E). Tissue homogenates were serially diluted in PBS and plated for viable counts as follows: BHI agar (BD-BBL, #211059) for total bacteria; Rogosa agar (Himedia, #M407) for lactobacilli; and Citrate Azide Tween Carbonate (CATC) base agar (Merk, #1.10279; supplements from Himedia, #FD235) for enterococci. Agar plates for bacterial recoveries were incubated anaerobically at 37°C for 3–5 days. To quantify *Candida*, tissue homogenates were plated in Sabouraud Dextrose (BD-Difco, #210950) agar with 10 µg/mL chloramphenicol (Sigma) and incubated aerobically at 37°C for 48–72 h.

#### Bacterial DNA extraction and qPCR

Tongue samples were incubated overnight using a custom lysis buffer (36) and were homogenized using zirconia beads (Ambion, USA) in a Fastprep-24 (MP Biomedicals, USA) to disrupt microbial cells. The following day, tongues were processed using the Qiagen DNA Blood and Tissue mini kit. DNA quantity and quality were evaluated using a NanoDrop. Total oral bacteria, lactobacilli (at genus level), enterococci (at genus level), *L. johnsonii* MT4 (at strain level), and fungal biomass were quantified using primers and amplification conditions as detailed in Table S1.

## RESULTS

### *L. johnsonii* is the dominant oral cultivable *Lactobacillus* in female C57BL/6 mice

**Fig 2:**
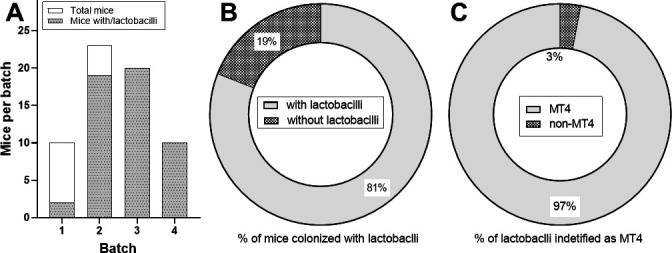
The most abundant cultivable *Lactobacillus* from the oral cavity of female C57BL/6 mice was molecularly identified as *L. johnsonii* strain MT4/NCK2677. The oral cavity of mice (*n* = 63) from four independent shipment batches was sampled using cotton swabs within 12 days of arrival. Swabs were cultured on Rogosa agar and incubated anaerobically at 37°C for 48–72 h. Bacterial cells were fragmented, and their DNA was amplified using *Lactobacillus* genus-level primers and strain-specific primers. Identity was confirmed *via* agarose gel electrophoresis (Fig. S1B). (**A**) *Lactobacillus* (genus level) colonization ranged from 20% to 100%. (**B**) In total, 81% of mice (51 out of 63) were colonized by cultivable *Lactobacillus sp*. (**C**) 97% of the cultivable oral lactobacilli were identified as *L. johnsonii* strain MT4/NCK2677.

*L. johnsonii* is a major colonizer in the oral cavity of C57BL/6 female mice and strain MT4 administration can induce global changes in the murine oral microbiome (17, 19). We thus hypothesized that MT4 may be a dominant oral strain in these mice. We conducted a comprehensive screening of the oral lactobacilli colonizing 63 C57BL/6 female mice from four independent batches (*n* = 10, 23, 20, and 10 mice per batch, respectively). Based on colony morphology, we selected 389 bacterial colonies from Rogosa agar plates for further analysis. In all, 347 colonies had a morphotype consistent with lactobacilli (Fig. S1A). Molecular identification, employing genus-specific and strain-specific DNA primers, validated *via* PCR-PAGE, showed that *Lactobacillus* colonization fluctuated across batches, with 20%, 83%, 100%, and 100% of mice colonized, respectively (Fig. 2A). These differences may be due to a variation in baseline oral communities among batches, or due to different sampling times within our 12-day window. It is also possible that oral swabbing of live mice has sensitivity and mouse-mouse reproducibility limitations in recovering oral bacteria since MRS colonies with *Lactobacillus* morphology are consistently recovered from excised tongues after necropsy (17, 19, and this work). Nevertheless, pooling data from all batches showed that most C57BL/6 female mice from this vendor (81%) were colonized by cultivable lactobacilli (Fig. 2B). Strain-specific primers amplified DNA from 335 out of the 347 *Lactobacillus* colonies (97%) indicating that *L. johnsonii* strain MT4/NCK2677 was present in all *Lactobacillus*-colonized mice (Fig. 2C; Fig. S1B). None of the 37 non-*Lactobacillus*-like colonies were identified as lactobacilli by PCR. We conclude that *L. johnsonii* strain MT4/NCK2677 is a dominant cultivable *Lactobacillus* strain within the oral mucosal microbiota of C57BL/6 female mice from Jackson Laboratories.

### Oral inoculation of *L. johnsonii* strain MT4 increases the oral *Lactobacillus* biomass in *Candida*-infected mice and mitigates the infection-induced enterococcal bloom

**Fig 3:**
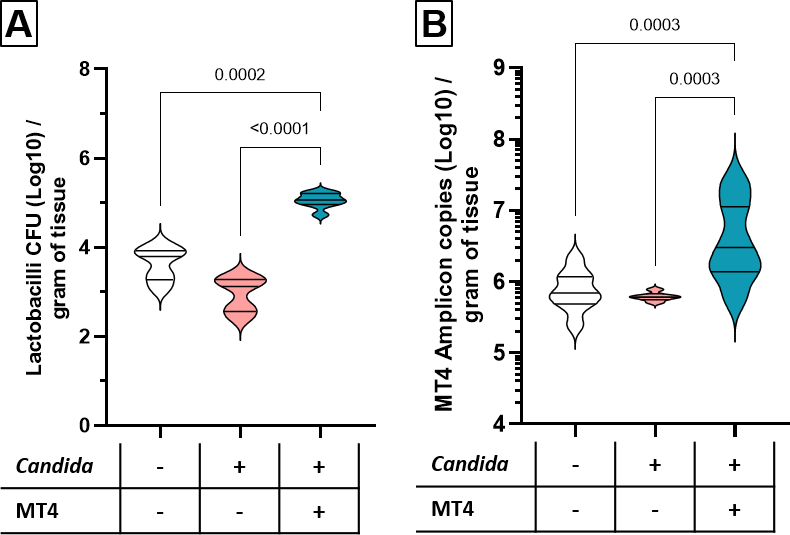
MT4 inoculation of *Candida*-infected mice increases the oral *Lactobacillus* biomass. Cortisone-immunosuppressed mice were co-infected with *C. albicans* and *L. johnsonii* strain MT4. Mice were euthanized on day 5 post-infection. Lactobacilli were quantified on mouse tongues *via* (**A**) colony counts on Rogosa agar and (**B**) strain-specific qPCR. Results are from 8 to 16 mice/group in two independent experiments. One-way ANOVA with uncorrected Dunn’s test.

*C. albicans* infection causes a severe reduction in the diversity of the murine oral mucosal bacterial microbiome (19), along with a reduction in resident oral lactobacilli populations (17, 33). Oral supplementation with strain MT4 restores a diet-induced reduction in oral lactobacilli in C57BL/6 mice (17). We thus asked whether inoculating strain MT4 in *Candida*-infected mice would similarly restore the oral mucosal *Lactobacillus* biomass. Indeed, inoculation with strain MT4 increased the viable counts of lactobacilli and the biomass of this strain on the oral mucosa, as shown with strain-specific qPCR (Fig. 3A and B).

**Fig 4:**
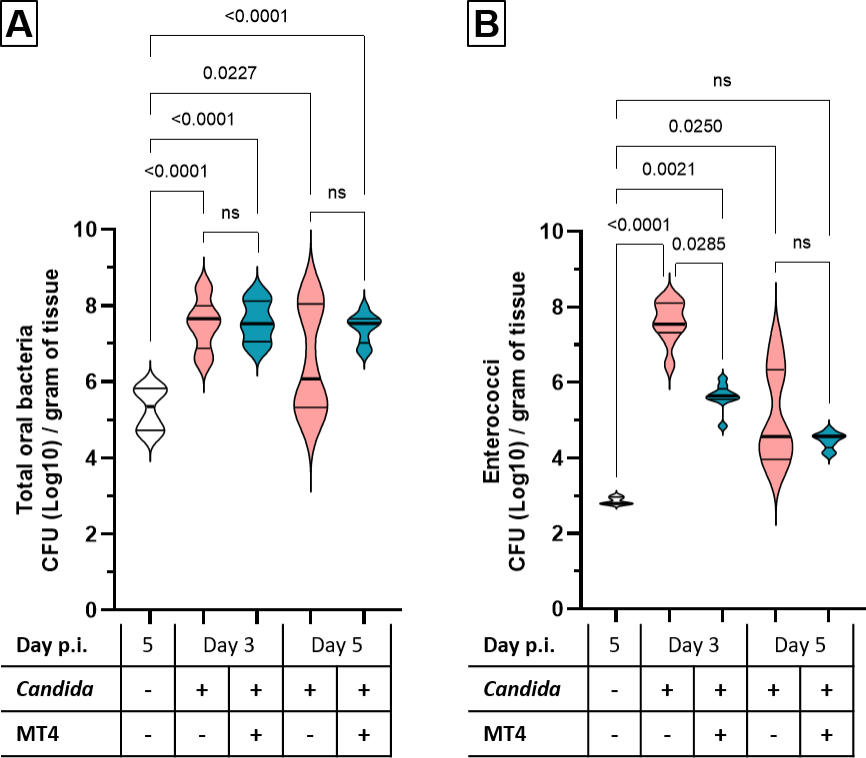
MT4 reduces the *Candida*-induced rise in indigenous enterococcal burdens. Cortisone-immunosuppressed mice were inoculated with *C. albicans* alone or in combination with *L. johnsonii* strain MT4. Mice were euthanized on day 3 or 5 post-infection. (**A**) Increased bacterial biomass in all infected groups as assessed by viable counts of tongue homogenates on BHI agar. (**B**) Significantly reduced viable counts of oral enterococci on day 3 in the MT4-inoculated OPC cohort as assessed by viable counts on selective CATC agar. Data from 8 to 16 mice per condition from two independent experiments. One-way ANOVA with the uncorrected Dunn’s test.

*C. albicans* oral infection is characterized by significant dysbiotic shifts in the bacterial microbiome, resulting in increased bacterial biomass, reduced diversity, and the expansion of specific bacterial taxa, most notably enterococci in the mouse OPC model (8, 33, 37). In mice with a perturbed oral microbiome, MT4 inoculation can reduce the relative abundance of enterococci (17). Based on this information, we next asked whether MT4 inoculation in *Candida*-infected mice can result in a reduction in the resident enterococcal populations. We found that there was a significantly increased bacterial biomass in the oral cavity in all *Candida*-infected mice, including those supplemented with strain MT4 (Fig. 4A). Consistent with prior reports (8, 33, 37), *Candida* mono-infection induced the expansion of resident enterococcal populations, which peaked on day 3 and declined by day 5 when recovery is ongoing in this model (33). Supplementation with strain MT4 significantly curtailed the increase in oral enterococcal populations on day 3 post-infection (Fig. 4B). This trend continued on day 5, although it did not reach statistical significance compared to the *Candida* mono-infected group. Together, these results show that MT4 impacts the oral microbial ecology in *Candida*-infected mice by increasing lactobacilli and reducing enterococci during fungal infection.

### *L. johnsonii* strain MT4 attenuates mucosal damage associated with *Candida* infection

**Fig 5:**
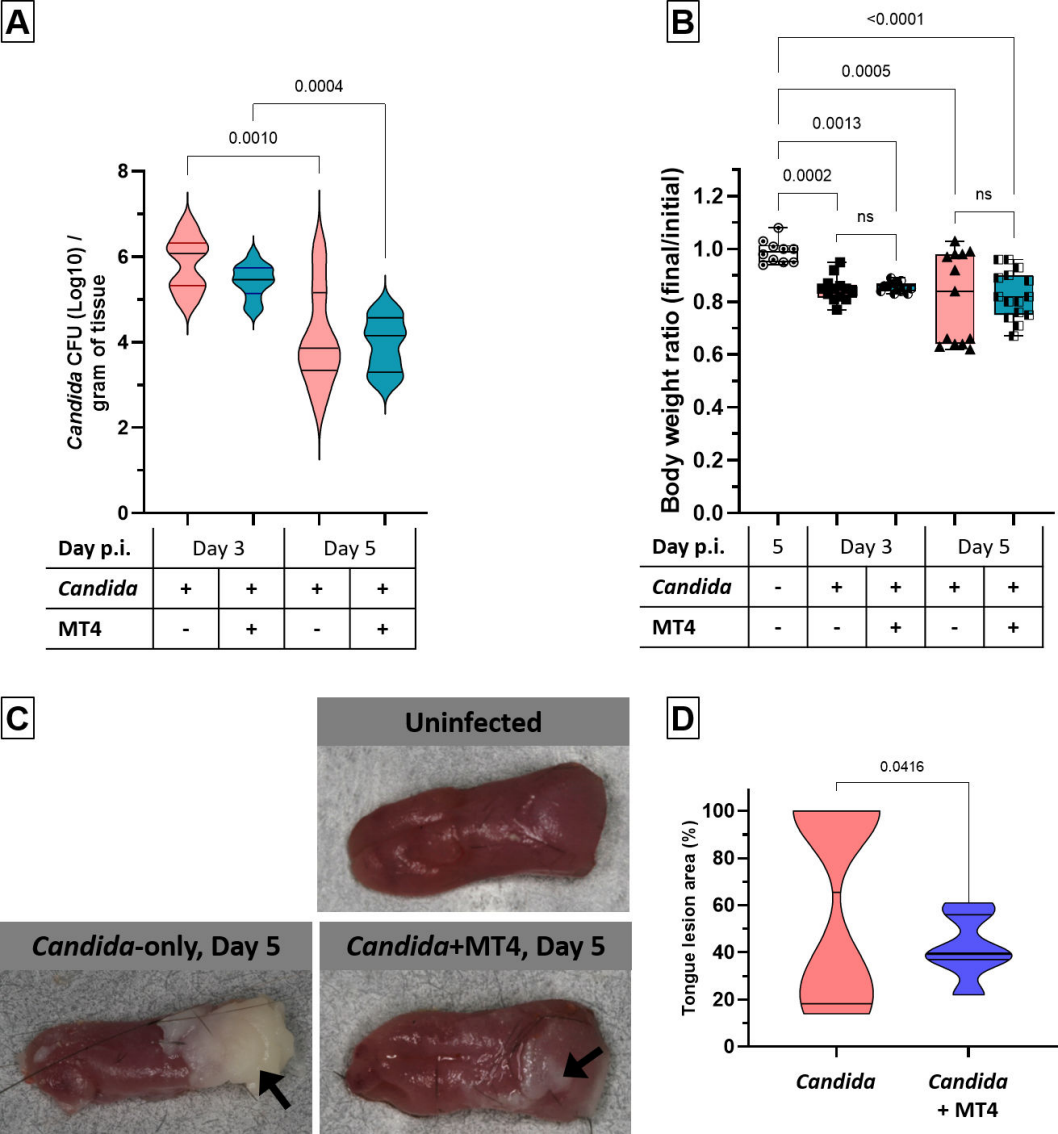
*L. johnsonii* strain MT4 reduces the severity of oral *C. albicans* lesions without significantly affecting fungal burdens. Cortisone-immunosuppressed mice were inoculated with *C. albicans* alone or in combination with *L. johnsonii* strain MT4. Mice were euthanized on day 3 or 5 post-infection. (**A**) Effect of strain MT4 inoculation on *C. albicans* burdens as assessed by viable counts of tongue homogenates on chloramphenicol-supplemented Sabouraud dextrose agar. (**B**) Body weight loss on days 3 and 5 post-infection, expressed as initial/final body ratio. (**C**) Macroscopic tongue images of mucosal biofilms (white patches) on day 5 pi. (**D**) Image J quantification of biofilm surface area on day 5 pi. Data from *n* = 8–16 mice per group in two independent experiments. One-way ANOVA with uncorrected Dunn’s test.

In a mouse model of OPC, we previously showed that the rise in *Enterococcus* populations during infection contributes to oral mucosal damage (8). Based on the reduction in enterococci (Fig. 4B) and the antifungal activity of the MT4 strain *in vitro* (21), we hypothesized that oral mucosal damage would be attenuated in the *Candida*-infected MT4-inoculated mice. We found a trend toward decreased viable *Candida* counts in the MT4-inoculated cohorts, particularly on day 3; however, this was not statistically significant (Fig. 5A).

Longitudinal monitoring of mouse body weight—as a marker of infection severity—did not demonstrate significant differences between infected groups, irrespective of MT4 inoculation. However, while weight loss did not progress in most *Candida*-infected mice of the MT4-inoculated group, a larger number of mice continued to lose weight in the non-inoculated group until day 5 (Fig. 5B). Consistent with this finding, tongue biofilm lesions were significantly reduced in the MT4-inoculated compared to the non-inoculated group on day 5 (Fig. 5C and D).

**Fig 6:**
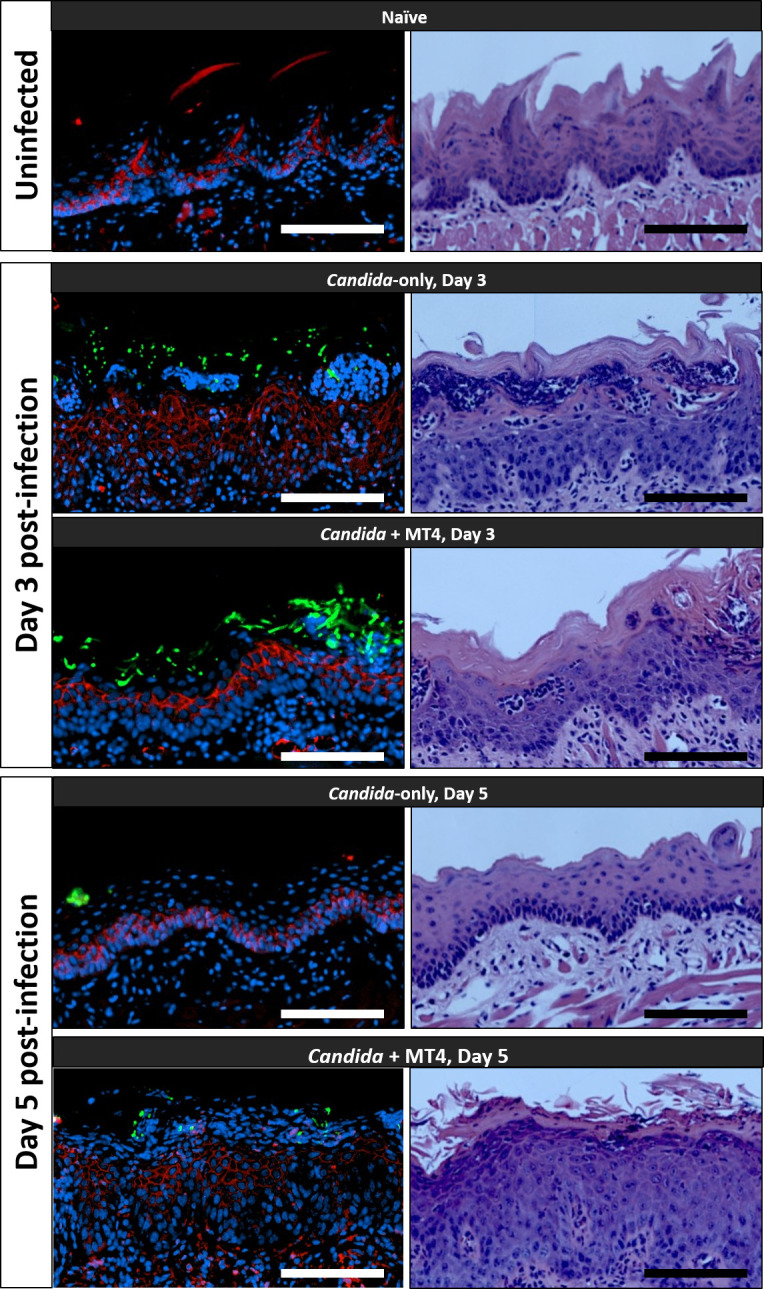
Histopathological analyses of *Candida*-induced disruption of the oral mucosa. Mice were infected with *C. albicans* with and without inoculation with *L. johnsonii* MT4, and tongues were excised and processed for histopathological analyses on days 3 and 5 post-infection. Left panel: Tongue tissue sections with immunofluorescence staining (*Candida*: FITC-conjugated anti-*Candida* antibody, green; nuclei: Hoechst 33258, blue; and E-cadherin: mouse a-E-cadherin antibody, red). Right panel: Hematoxylin-eosin staining. Representative tissue sections from 2 to 3 mice/group in two experiments. Scale bar = 100 µm.

Histopathological examination of the structural integrity of the oral mucosa in the two *Candida*-infected groups showed that on day 3 in *Candida* mono-infected mice, there was significant thinning of the keratin layer and a pronounced intraepithelial inflammatory infiltrate. In comparison, in MT4-inoculated mice, the keratin layer appeared to be partially intact with a few inflammatory intraepithelial microabscesses. On day 5, *Candida*-mono-infected mice had lost the entire keratin layer and continued to have significant epithelial erosion, while the MT4-inoculated mice had a significantly thicker, parakeratinized oral epithelium on the entire dorsal tongue surface (Fig. 6; Fig. S2). Because MT4 reduced enterococci (Fig. 4B), which can contribute to the dissolution of epithelial E-cadherin from cell junctions during fungal infection (8), we also evaluated the integrity of the mucosal barrier by assessing the immunoreactivity pattern of E-cadherin (38). Both *Candida*-infected groups exhibited a reduction in the E-cadherin fluorescent signal on day 3, although we observed a stronger signal in the basal epithelial layer in the MT4 coinfected group. By day 5 in the MT4-inoculated group, E-cadherin immunoreactivity was present throughout the thick epithelial layers, whereas in the *Candida* alone group, the E-cadherin signal was restricted to the basal layer (Fig. 6). Collectively, these findings suggested that strain MT4 attenuates mucosal damage and may expedite lesion resolution in this OPC model.

### Strain MT4 inhibits fungal metabolic activity, and chitinase activity contributes to this inhibition

**Fig 7:**
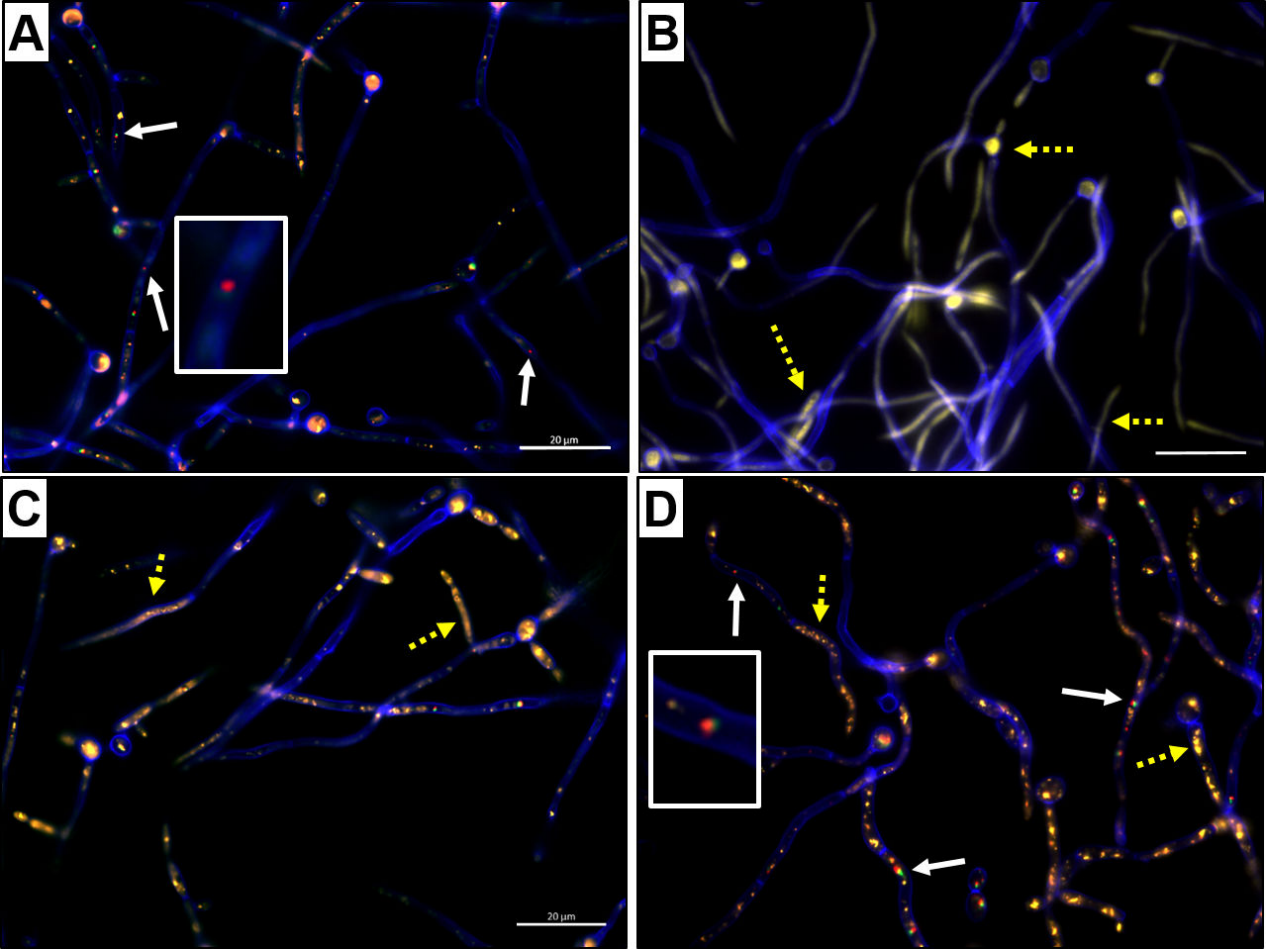
*L. johnsonii* strain MT4 inhibits fungal metabolic activity, which is enhanced when in physical contact with *C. albicans* cells. *C. albicans* was cocultured with *L. johnsonii* strain MT4 in BHI broth, aerobically in 5% CO_2_ at 37°C for 4 h, either in direct contact or separated by placing MT4 on transwell insert filters. Fungal metabolic activity was assessed *via* the fluorescent stain FUN-1 and cells were counterstained with Calcofluor White. (**A**) Production of fluorescent red CIVS (white arrows) in metabolically active *Candida* monocultures stained with FUN-1 (shown in detail in the inset). (**B**) Heat-killed *C. albicans*. The green-yellow intracellular fluorescence indicates that cells are metabolically inactive (yellow arrows). (**C**) MT4 and *C. albicans* 4 h cocultures. Hyphae have fewer CIVS, with many cells instead showing green-yellow intracellular fluorescence. (**D**) MT4 and *C. albicans* cocultured with a physical barrier. While many hyphae still show green-yellow intracellular fluorescence, there is an increased number of CIVS compared to (**C**). Representative images from one of two independent experiments. Scale bar: 20 µm.

Strain MT4 has significant anti-*Candida* activity *in vitro* (21). To follow up on this work, we further mechanistically explored this activity. In cocultures, fungal and bacterial cells formed closely associated coaggregates within the first 30 min of incubation (Fig. S3). Physical interactions of *C. albicans* with other bacterial species [e.g., *Acinetobacter baumannii* (39) or *L. rhamnosuss* GG (40)] have detrimental effects on fungal growth; thus, we further hypothesized that the antifungal activity of strain MT4 is contact-dependent. We employed the FUN-1 cell-permeant stain, which facilitates the visualization of metabolically active fungal cells through the production of red fluorescent cylindrical intravacuolar structures (CIVS) (Fig. 7A). Metabolically inactive or non-viable organisms, such as heat-killed hyphae, lack CIVS and display a green-yellow fluorescence (Fig. 7B). *C. albicans* cocultured with strain MT4 had a reduction in the presence of CIVS, coupled with an increase in green-yellow fluorescence, indicating that strain MT4 decreased the fungal metabolic activity (Fig. 7C and D). This decrease was more pronounced when the fungal and bacterial cells were in physical contact (Fig. 7C) compared to when a physical barrier separated them (i.e., MT4 placed in a filter insert) (Fig. 7D). This observation suggests that the MT4 activity is associated with bacterial products that have direct anticandidal properties. These products may be cell-surface associated or locally more concentrated and thus more effective when bacterial cells are in physical proximity to *C. albicans*. Collectively, our findings agree with previous observations that *Lactobacillus* species co-aggregate with *C. albicans* and that physical interaction is important in their antifungal activities (21, 40–42).

**Fig 8:**
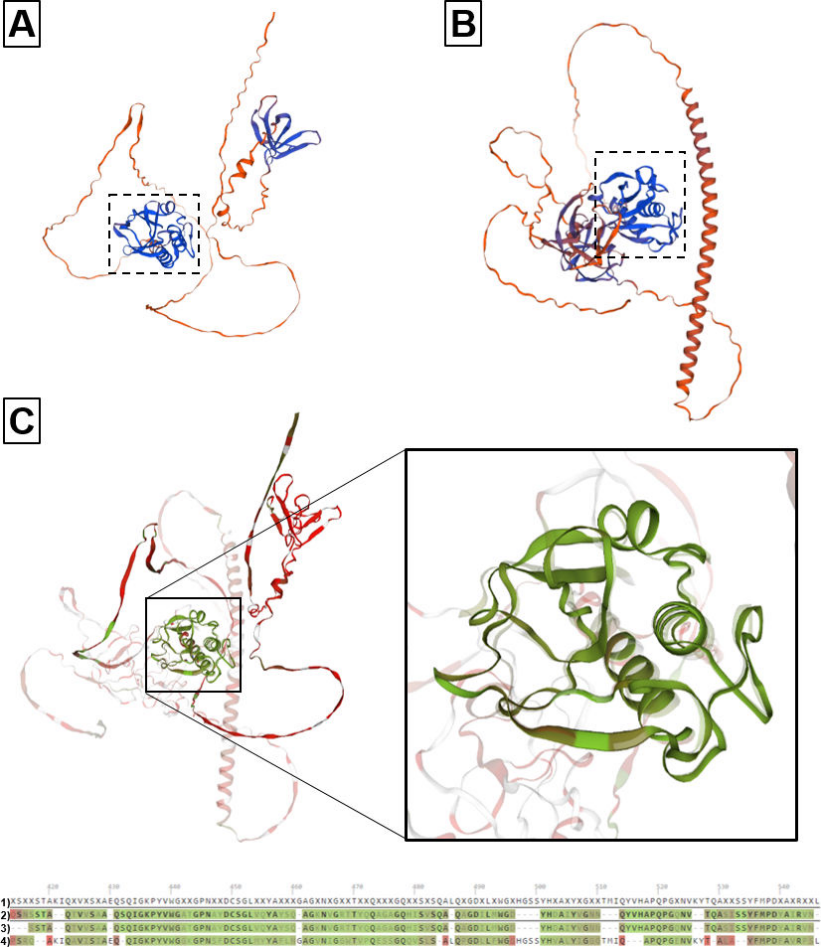
The 3D structure of (**A**) a strain MT4 putative chitinase and (**B**) Msp1/p75 (a peptidoglycan hydrolase with chitinase activity) was predicted using their protein sequence from UniProt and modeled in the Swiss model. The dotted square in A and B shows the location of the NlpC/P60 domain, a chitin-binding domain found in proteins with chitinase-like activity. (**C**) These gene products were superimposed in the Swiss model (**C**), confirming they share the NlpC/P60 domain. The paired sequences at the bottom (**C**) show the peptide region corresponding to the NlpC/P60 domain [1 = consensus sequence, 2 = MT4 putative chitinase, 3 = NlpC/P60 domain sequence, and 4 = Msp1/p75 (UniProt A0AA43SZT2)].

*L. johnsonii* strain MT4 has a gene encoding for a putative chitinase (peptidoglycan hydrolase, GenBank: MCF1582664.1) (20, 21). This MT4 gene product shares 49.17% homology to Msp1/p75 (UniProt: A0AA43SZT2, E value 7e-32, *via* TBLASTN), a peptidoglycan hydrolase with known chitinase-like activity (40). Because the protein function cannot be easily extrapolated from its sequence and assessing the relationship between homology and function is challenging (43), we modeled the 3D structure of the putative chitinase from this strain and the peptidoglycan hydrolase Msp1/p75 using the Swiss model (Fig. 8A and B) and AlphaFold. We found that both this MT4 putative chitinase and Msp1/p75 contain the chitin-binding NlpC/P60 domain, also found in other hydrolases with chitinase-like activity (44), supporting the potential for enzymatic function. Next, we superposed the NlpC/P60 domain sequence to this MT4 putative chitinase and Msp1/p75 from other lactobacilli (UniProt A0AA43SZT2, A0A7D9N840, and A0A9 × 4XB52), and confirmed that the NlpC/P60 domain 3D structure is shared between these products (Fig. 8C). Further MT4 genome exploration revealed that a putative glucanase (glycoside hydrolase family 73, Genbank: MCF1583094.1), with a transmembrane domain, is homologous to a chitinase from *Lactiplantibacillus plantarum* (UniProt: A0A1E3KVC2; 36.26% identity, E value 1e−22, TBLASTN). A comparison of their predicted 3D conformation shows that these products share structural similarities (Fig. S4). Together, these findings support the presence of chitinase activity in strain MT4.

**Fig 9:**
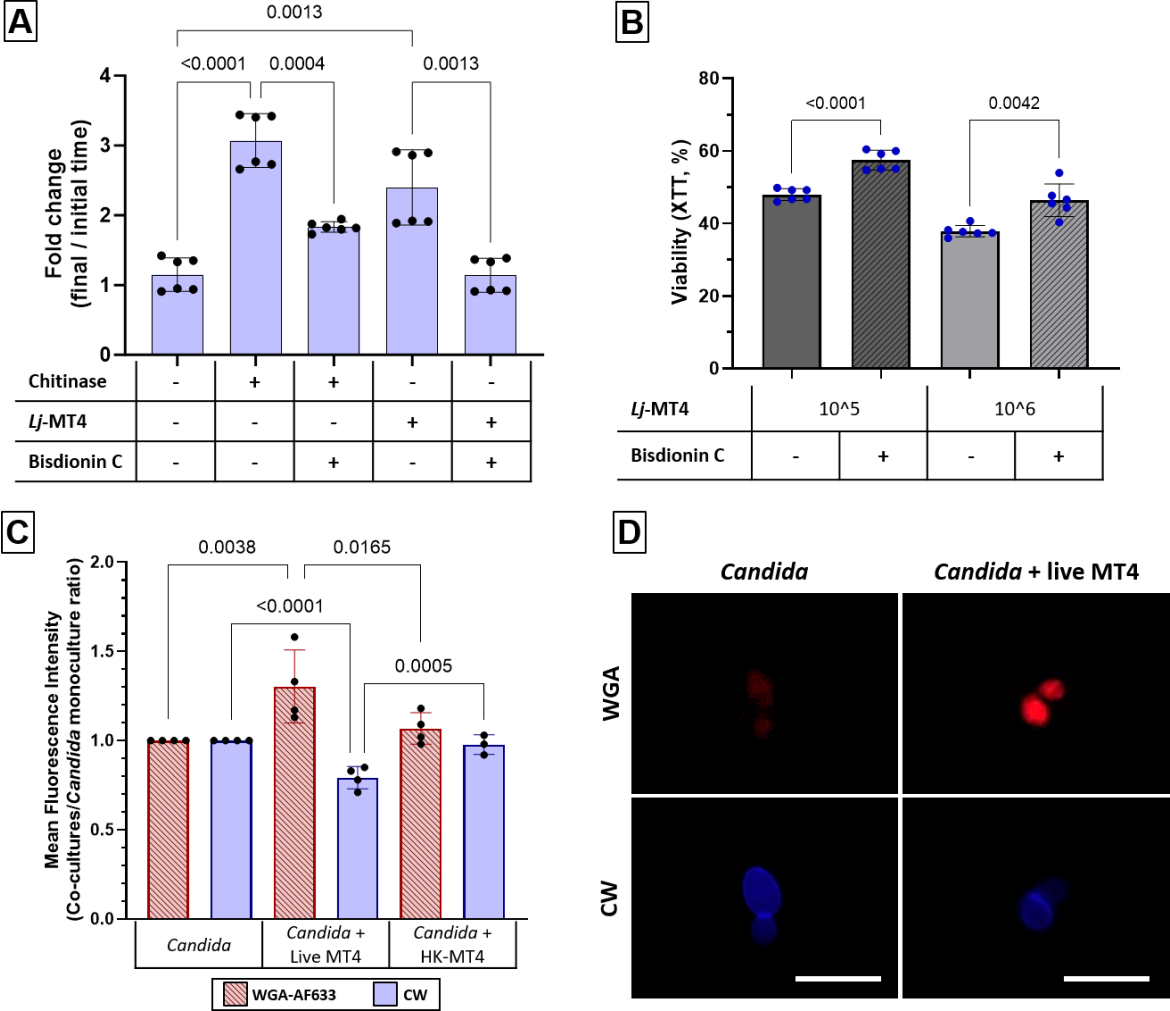
*L. johnsonii* strain MT4 exhibits chitinase activity, which is partially responsible for its antifungal properties. (**A**) Chitinase activity colorimetric assay based on chitin-azure breakdown by incubating MT4 anaerobically overnight in MRS broth without dextrose containing 0.3% m/v chitin-azure. Chitinase and Bisdionin C were used as positive and negative controls, respectively. Chitinase activity was assessed by measuring the azure dye released, *via* spectrophotometry. MT4-conditioned media significantly increased the breakdown of the chitin-azure substrate, which was inhibited by the chitinase inhibitor Bisdionin C. (**B**) *C. albicans* was cocultured with MT4, with and without Bisdionin C, in BHI broth, aerobically with 5% CO_2_ at 37°C for 4 h. Fungal metabolic activity was assessed using the XTT assay. MT4 significantly reduced fungal metabolic activity, and Bisdionin C partially protected *C. albicans* from this effect. (**C**) Paraformaldehyde-fixed *C. albicans* yeast cells were incubated with strain MT4 overnight in MRS without dextrose. Heat-killed MT4 was used as negative control. Fungal cells were stained with 0.5% wheat germ agglutinin-Alexa Flour 633 fluorescent conjugate (WGA-AF633) which binds to chitin residues, and counterstained with the chitin-binding CW. Mean fluorescence intensity from *n* = 20,000 *Candida* cells per experimental condition *via* FACS. (**D**) Representative fluorescence images from two independent experiments show that the WGA-AF633 signal was increased while the CFW signal decreased in the live MT4-treated *Candida* cells. Scale bar = 10 µm. **A**–**C**: Results are from two independent experiments each with 2–3 technical replicates. One-way ANOVA with the uncorrected Dunn’s test.

To confirm the presence of MT4 enzymatic activity, we used a colorimetric assay with chitin linked to the Remazol Brilliant Blue dye (chitin-azure) as substrate. Upon cleavage by chitinases, chitin-azure releases the dye measured *via* spectrophotometry, which serves as a quantitative indicator of enzymatic activity. Strain MT4 displayed significant chitinase activity, as evidenced by a marked increase in the absorbance levels in supernatants of overnight cultures grown in the presence of chitin-azure, which was inhibited by the chitinase inhibitor Bisdionin C (Fig. 9A).

Next, we asked whether chitinase activity played a role in inhibiting the fungal metabolic activity by strain MT4 in the 4-h coculture system. Using the XTT assay, we confirmed that strain MT4 reduced the metabolic activity of *C. albicans* in a dose-dependent manner and found that Bisdionin C partially but significantly protected fungal cells (Fig. 9B), indicating that chitinase is one factor directly responsible for antifungal activity.

Based on these results, we reasoned that chitinase from strain MT4 may compromise the integrity of the fungal cell wall. To test this, we stained fungal cells with the fluorescent conjugate wheat germ agglutinin-Alexa Flour 633 (WGA-AF633) and the chitin-binding dye CW (45). WGA is a protein that binds specifically to internal N-acetylated sugar residues exposed by chitinases (32). Changes in the fluorescence intensity of both WGA-AF633 and CW can indicate changes in the chitin content of the fungal cell wall. To rule out the possibility that the chitinase activity originated from *Candida*, paraformaldehyde-fixed yeasts were used in these assays. Consistent with this idea, live MT4 organisms significantly increased the WGA-AF633 and reduced the CW fluorescent signals on the fungal cell surface (Fig. 9C). Heat-killed MT4 did not significantly affect WGA-AF633 binding, showing that live bacteria with intact enzymatic activity are required for this effect. Fluorescence microscopy images are consistent with the FACS data, showing that live MT4 organisms reduced the CW and increased the WGA-AF633 fluorescent signals on the surface of fixed yeast (Fig. 9D).

**Fig 10:**
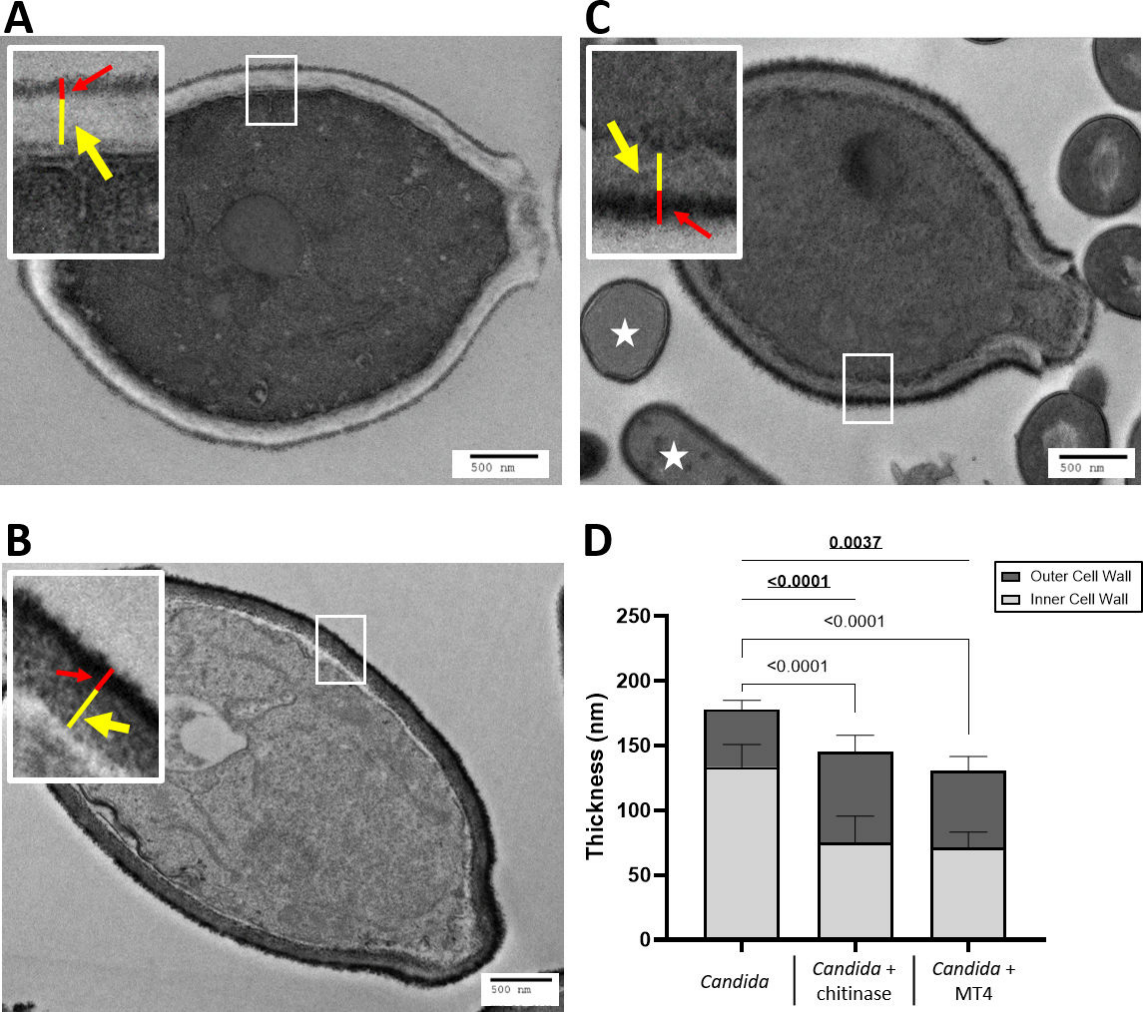
Disruption of the fungal cell wall ultrastructure by strain MT4. To assess the impact of MT4 on the fungal cell wall ultrastructure *via* TEM, paraformaldehyde-fixed *C. albicans* cells were cocultured with MT4 overnight in MRS without dextrose. Untreated and chitinase-treated cells were used as negative and positive controls, respectively. *L. johnsonii* strain MT4 disrupts the integrity of the fungal cell wall, as seen by TEM. Paraformaldehyde-fixed yeast cells (**A–C**): (**A**) untreated control, (**B**) exposed to chitinase, or (**C**) live MT4 overnight. The thickness of the outer (red lines and arrows) and inner (yellow lines and arrows) cell wall layers were quantified by Image J. (**D**) Chitinase and MT4 (white stars, in panel C) significantly reduced the thickness of the inner cell wall, suggesting a collapse of the cell wall from the reduction in the integrity of chitin. By contrast, the outer cell wall thickness increased, as shown by the presence of long mannoprotein fibrils extending outwards, suggesting additional disruptions of the fungal cell wall ultrastructure. The significance values in underlined bold numbers are from the outer cell wall, while the regular font values are from the inner cell wall. Four locations per cell were measured and averaged. Ten cells per condition from one experiment were measured. Scale bar = 500 nm. One-way ANOVA with Welch’s correction.

To complement these findings, we evaluated the effect of MT4 on the ultrastructural integrity of the fungal cell wall of *C. albicans via* TEM. *C. albicans* cell wall has an electron-dense outer layer that is formed by mannoproteins and an inner layer of low electron density composed of β (1–3)- and β (1–6)-glucan (46), contiguous to the cell membrane. Chitin microfibrils are dispersed throughout the inner cell wall (47). TEM analysis revealed that while *C. albicans* exposed to either chitinase or MT4 retained the two distinct cell wall layers, these treatments significantly reduced the thickness of the inner cell wall by approximately 45%, from 133.3 nm ±16.8 nm in untreated cells to 75.3 nm ±19.3 nm and 71.4 nm ±11.2 nm in the chitinase- and the MT4-treated cells, respectively. Since we used non-viable paraformaldehyde-fixed *Candida* cells in this assay, it is reasonable to assume that the decrease in thickness is due to an MT4-induced collapse of the inner cell wall caused by reduction in the chitin content (Fig. 10). In the chitinase- and the MT4-treated *Candida* cells, the inner wall appeared more electron-dense (darker contrast) than in the untreated cells, possibly due to further biochemical changes in the cell wall. Interestingly, the outer cell wall thickness was significantly increased from 44.8 nm ±6.5 nm in the untreated cells to 69.9 nm ±12.1 nm and 59 nm ±10.6 nm in the chitinase- and the MT4-treated cells, respectively. This suggested that more mannoprotein fibrils were free to extend outwards from the external border of the cell wall (Fig. 10). Overall, these findings show a disruption of the fungal cell wall ultrastructure by strain MT4, beyond a single effect on chitin.

**Fig 11:**
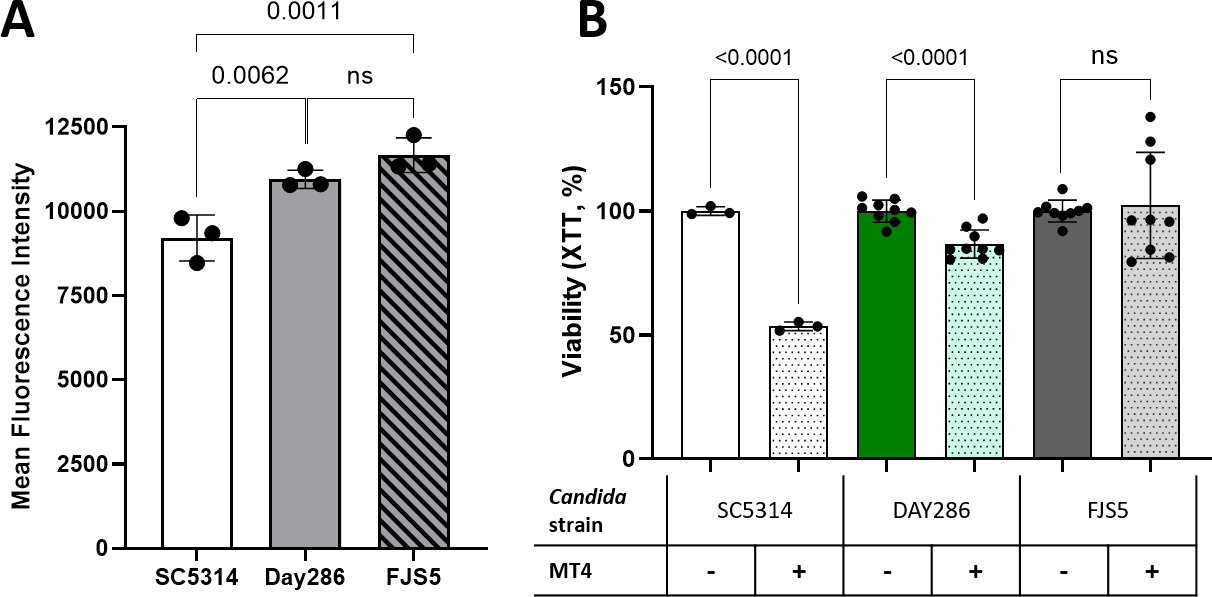
Chitin content influences *C. albicans* susceptibility to MT4. (**A**) *C. albicans* strains SC5314, DAY286 and its isogenic *cht2/cht2* mutant strain FJS5 were stained with CW. Chitin content as assessed by FACS analysis of CW binding. Strains DAY286 and FJS5 have higher CW binding than strain SC5314, indicating higher cell wall chitin content. Each dot represents the mean MFI of 20,000 cells from three replicates. (**B**) *C. albicans* strains SC5314, DAY286, and FJS5 were cocultured with MT4 in BHI broth, aerobically with 5% CO_2_ at 37°C for 4 h, and fungal viability was assessed by the XTT assay. Strains with higher chitin cell wall content (DAY286 and FJS5) were less susceptible to MT4. Data from 2 to 3 independent experiments with technical replicates. One-way ANOVA with the uncorrected Dunn’s test.

We next hypothesized that strains with higher chitin content in the cell wall would be less susceptible to MT4 antifungal activity. To test this hypothesis, we analyzed CW binding to *C. albicans* strains SC5314 (wildtype), DAY286 (reference), and FJS5, a DAY286-derived *cht2/cht2* mutant strain by FACS. We reasoned that the chitinase mutant *C. albicans* FJS5 might exhibit higher levels of chitin due to lower levels of chitinase activity. As predicted, strain FJS5 had the highest CW binding, suggesting a higher amount of chitin in the cell wall (Fig. 11A). In addition, the effect of *L. johnsonii* strain MT4 on the metabolic activity of the three *C. albicans* strains was inversely correlated with their chitin content, with the fungal strain FJS5 being entirely resistant (Fig. 11B). These results were confirmed with an additional set of *cht2/cht2* mutant and reference strains (*C. albicans* strains SN670 –wildtype- and SN250 –*cht2/cht2* mutant*-*, data not shown). Taken together, our results indicate that *L. johnsonii* strain MT4 chitinase activity damages the fungal cell wall and compromises fungal viability.

## DISCUSSION

Lactobacilli represent a major component of the murine oral bacterial microbiome (17, 33, 48). In this work, we discovered that the vast majority of the cultivable oral lactobacilli in C57BL/6 mice are likely represented by a single *L. johnsonii* strain. Although we did not quantify the abundance of strain MT4 in the lower GI tract, it is possible that it colonizes the entire GI tract since an almost identical strain (NCK2677) was isolated from fecal samples of the same mouse genetic background (26). The oral mucosa is a nutrient-rich environment that may prevent niche partitioning, causing closely related lactobacilli species to compete for nutrients, resulting in one *Lactobacillus* strain becoming dominant (49). Strain dominance across species is also a characteristic of the human oral (50) and gut (51) microbiomes. Our finding aligns with the observation that *L. johnsonii* is the dominant species in the stomach of C57BL/6 female mice from the same vendor (52).

A study in C57BL/6 mice from two different vendors (Taconic and JAX) showed that once the oral bacterial microbiome is established after 4 weeks of age, its structure remains largely stable (48). However, we previously showed that oral *Lactobacillus* communities can be perturbed by *Candida* infection, or dietary changes (17, 19). This study supports the hypothesis that strain MT4 may play an important role in restoring the bacterial community shifts caused by oral infection. In our OPC model, strain MT4 reduced enterococcal burdens early in the infection process. Similarly, in a *C. glabrata* mouse colitis model, a fecal *L. johnsonii* isolate contributed to reducing both fungal and enterococcal burdens, leading to decreased inflammation and histopathology (53). *In silico* genome sequence analysis of the MT4 strain suggested the production of bacteriocins and biosurfactants (21), which could elicit direct anti-enterococcal activity. We also reported that strain MT4 has anti-enterococcal activity *in vitro* (19). The reduction in the *Enterococcus* burdens in the OPC model is important since these bacteria have been hypothesized to have a symbiotic relationship with *Candida* (54, 55) and under certain host environmental conditions can contribute to mucosal damage (8).

In the OPC model, an important observation was that strain MT4 increased the tongue epithelial thickness and reduced the severity of oral lesions, suggesting a role in strengthening the mucosal barrier against fungal infection. Since fungal burdens were not significantly reduced in the MT4-inoculated mice, this effect may be secondary to a reduction in enterococci with synergistic virulence (8). Failure of strain MT4 to cause a significant reduction in fungal burdens was unexpected since a diet-induced *L. johnsonii* enrichment in the oral mucosa has been associated with a reduction in fungal burdens in *Candida*-infected mice (19). However, oral bacteria exist and act in consortia, and inoculation with a single bacterial strain is unlikely to recapitulate global changes induced by diet. In addition, we and others have shown that bacteria can alter fungal virulence by influencing the host response without a significant effect on *Candida* biomass (8, 10, 56). Consistent with our findings, in a gut *C. albicans* infection model *L. rhamnosus* protection of the epithelial barrier was independent of an effect on fungal burdens (56). Along these lines, tissue-regenerative activities of lactobacilli have been reported across different mucosal sites both *in vivo* and in cell culture models *in vitro*. For instance, *L. crispatus* expedited the re-epithelialization of the MS74 vaginal cell line (57); *L. plantarum* fostered the regeneration of colon crypts and reduced inflammation in a colitis murine model (58); *L. rhamonosus* GG stimulated intestinal stem cell regeneration (59); and *L. johnsonii* protected the vaginal epithelial mucosal structure in a rat model of vulvovaginal candidiasis (60). *Lactobacillus* species may also promote tissue repair processes by regulating the expression of tight junction proteins, for example, zonula occludens-1 and occludin (59, 61).

In previous work, *in silico* genome analysis suggested that strain MT4 anti-*Candida* activity could be partially due to the production of chitinases (21), yet this mechanism of action remained to be elucidated. In this report, we analyzed the sequence and 3D structure of putative chitinases from strain MT4, including one homologous to Msp1/p75, a peptidoglycan hydrolase with chitinase-associated antifungal activity (40). We found that the putative MT4 chitinase gene has high homology to this hydrolase and shares the chitin-binding NlpC/P60 protein domain found in other hydrolases with chitinase-like activity. Our experimental data confirmed that strain MT4 has chitinase activity, which contributes to its *in vitro* antifungal action. Chitinase-associated antifungal activity against *Candida glabrata* has also been reported with a mouse fecal isolate of *L. johnsonii* (53). In *L. rhamnosus*, Msp1/p75 is localized at the poles of *Lactobacillus* cells (40). Based on our *in silico* genome analysis (20) and since this peptidoglycan hydrolase is conserved across *Lactobacillus* species (62), it is reasonable to assume that strain MT4 chitinase activity is due to this cell surface-associated hydrolase. This also explains the fact that the antifungal activity was at least partially fungal-bacterial cell contact-dependent. However, in our 4-h coculture system, chitinase activity was only partially responsible for the antifungal action of strain MT4. This, in conjunction with the fact that MT4-induced changes extended from the chitin-rich inner throughout the outer layer of the yeast cell wall, raises additional possible mechanisms of antifungal action for strain MT4. One such possibility is that a glucanase (a glycoside hydrolase family 8, GenBank: MCF1582481.1) encoded in the MT4 genome that can lyse O-glycosyl compounds (63), such as those found in *C. albicans* mannoproteins (64), is additionally responsible for yeast cell wall damage (20, 21). Because strain MT4 did not cause a significant reduction in fungal burdens in the OPC model, the chitinase activity we observed *in vitro* may not play a major role in the protection of the epithelial barrier *in vivo*. However, reduction in the cell wall chitin content by this and other chitinases is an important finding since it may increase fungal cell susceptibility to current antifungal therapies such as echinocandins (65).

Lastly, *L. johnsonii* MT4 is closely related to strain NCC533/La1 (20), a well-studied probiotic strain with therapeutic potential in human metabolic disorders (66), suggesting that it may have potential oral therapeutic applications yet to be explored. Further research is needed to determine whether MT4 can improve current antifungal therapies by direct interaction with *Candida* and/or by modulating host mucosal responses. Our study highlights the potential of *L. johnsonii* MT4 as a novel therapeutic agent for OPC. By enhancing our understanding of the role of this strain in the oral microbiome and its interactions with *Candida* and the host, we can contribute to the development of microbiome-based strategies to combat this infection.

## SUPPLEMENTAL MATERIAL

Figure S1 mbio.02416-24-s0001.tif *Lactobacillus* colony identification.

Figure S2 mbio.02416-24-s0002.tif Histopathology of mouse tongue.

Figure S3 mbio.02416-24-s0003.tif *Candida-Lactobacillus* coaggregation.

Figure S4 mbio.02416-24-s0004.tif Predicted 3D structure of putative chitinase.

Legends mbio.02416-24-s0005.docx Supplemental figure legends.

Table S1 mbio.02416-24-s0006.docx PCR primers.

ASM does not own the copyrights to Supplemental Material that may be linked to, or accessed through, an article. The authors have granted ASM a non-exclusive, world-wide license to publish the Supplemental Material files. Please contact the corresponding author directly for reuse.
