# Supplementary figures and images for: Lactobacillus johnsonii is a dominant Lactobacillus in the murine oral mucosa and has chitinase activity that compromises fungal cell wall integrity
Source: mBio. 2024 Sep 17;15(10):e02416-24. doi: 10.1128/mbio.02416-24 (PMC11481578; doi:10.1128/mbio.02416-24)

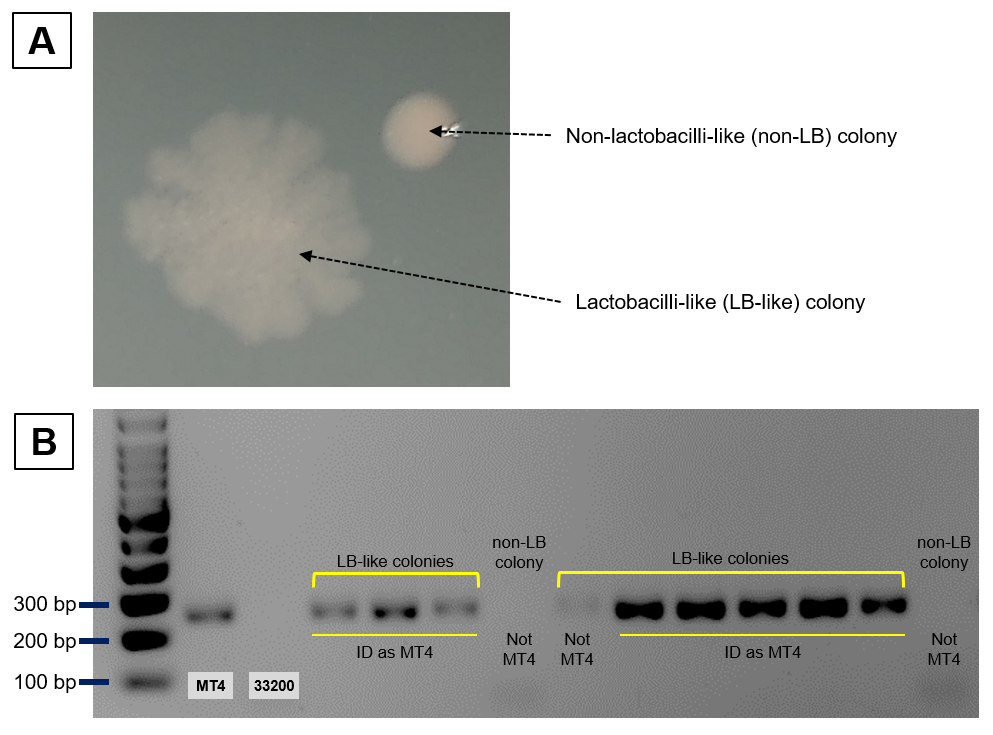

Supplement: Figure S1 — Lactobacillus colony identification. [file mbio.02416-24-s0001.tif]

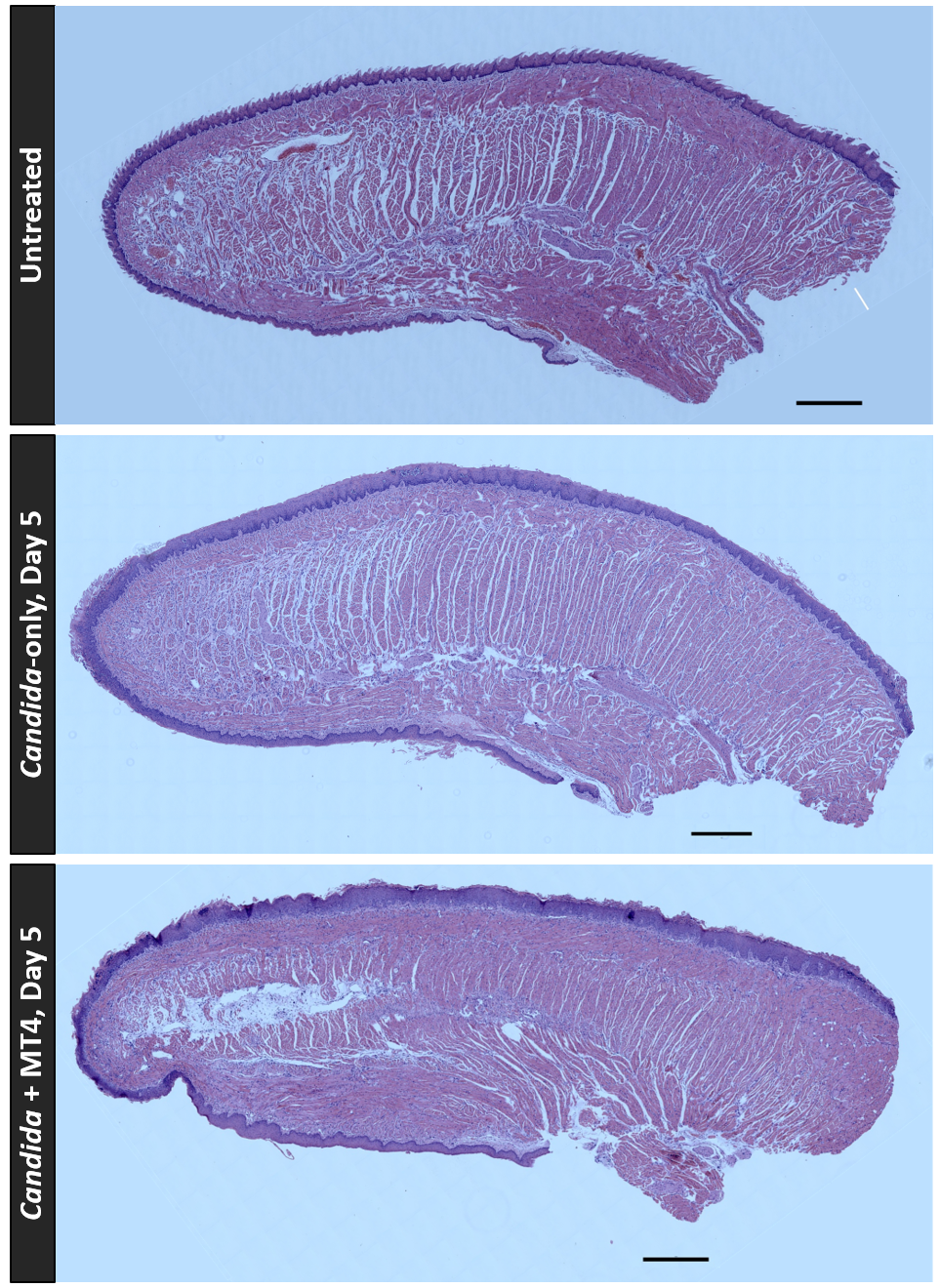

Supplement: Figure S2 — Histopathology of mouse tongue. [file mbio.02416-24-s0002.tif]

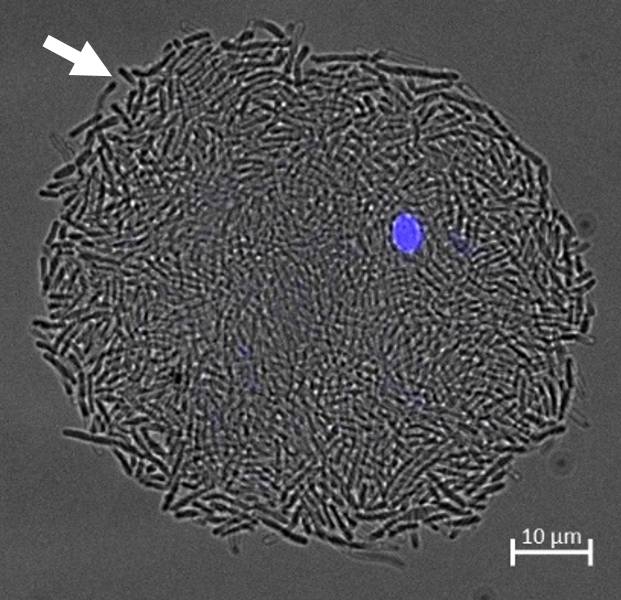

Supplement: Figure S3 — Candida-Lactobacillus coaggregation. [file mbio.02416-24-s0003.tif]

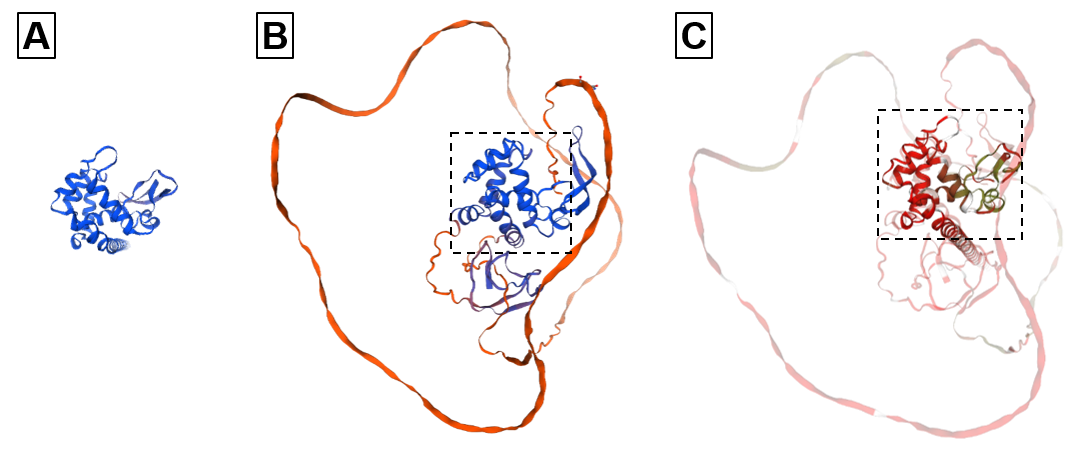

Supplement: Figure S4 — Predicted 3D structure of putative chitinase. [file mbio.02416-24-s0004.tif]
