# Supplementary material for: Lactobacillus johnsonii is a dominant Lactobacillus in the murine oral mucosa and has chitinase activity that compromises fungal cell wall integrity
Source: mBio. 2024 Sep 17;15(10):e02416-24. doi: 10.1128/mbio.02416-24 (PMC11481578; doi:10.1128/mbio.02416-24)
Supplement: Legends — Supplemental figure legends. [file mbio.02416-24-s0005.docx]

**Supplemental Legends**

**Supplementary Figure S1**. Morphology of the bacterial colonies plated in Rogosa agar. (A) Typical lactobacilli-like colonies display an irregular form with undulated margins, umbonate/flat elevation, and a pale white/yellow color. Selected non-lactobacilli-like colonies were circular, raised/convex, with entire margins, bright white color, and slight variations in shape and color (from pale cream to bright white). (B) Lactobacilli identities were confirmed via PCR and gel electrophoresis. DNA from *L. johnsonii* strains MT4 and ATCC 33200 were used as the positive and negative control, respectively, for the strain-specific primers.

**Supplementary Figure S2**. *Candida*-infected mice with and without MT4 oral supplementation 5 days post-infection. Whole tongue tissue sections stained with H&E show that the epithelial tissue in mice receiving MT4 was thicker throughout the dorsal surface than in mice inoculated only with *Candida*. Representative images from 2 independent experiments. Scale bar = 500 μM.

**Supplementary Figure S3.** *L. johnsonii* strain MT4 (10^6 cells/mL) was cocultured overnight with *C. albicans* (10^5 yeast cells/mL) in BHI broth, aerobically in 5% CO_2_ at 37 °C. When cocultured together, *L. johnsonii* strain MT4 (rod-shaped cells, white arrow) co-aggregates with *C. albicans* (in blue, stained with Calcofluor White). Scale bar: 10 um.

**Supplementary Figure S4.** The predicted 3D structure of (A) a second MT4 putative chitinase (Genbank: MCF1583094.1) and (B) a putative chitinase from *Lactiplantibacillus plantarum* (UniProt A0A1E3KVC2) using UniProt. (C) These products were superimposed in Swiss-model, confirming their structural similarities in their predicted 3D conformation.
