# Supplementary material for: Lactobacillus johnsonii is a dominant Lactobacillus in the murine oral mucosa and has chitinase activity that compromises fungal cell wall integrity
Source: mBio. 2024 Sep 17;15(10):e02416-24. doi: 10.1128/mbio.02416-24 (PMC11481578; doi:10.1128/mbio.02416-24)
Supplement: Table S1 — PCR primers. [file mbio.02416-24-s0006.docx]

**Supplemental Table S1**

| **Table S1.** Primers used in this work. | | | | |
| --- | --- | --- | --- | --- |
| **Primer set** | | **Sequence 5’ - 3’** | **Product size (bp)** | **Reference** |
| *Lactobacillus*, genus-level | (**F**) R16-1 | CTTGTACACACCGCCCGTCA | 209 - 250 | Dubernet et al. 2002 |
|  | (**R**) LbLMA1-rev | CTCAAAACTAAACAAAGTTTC |  |  |
| MT4 / NCK2677  strain-specific | (**F**) MT4_on49_F | TGAAGTCTGTTGATCCAGGTGT | 233 | This study |
|  | (**R**) MT4_on49_Rv | AAGGGGACGCCCTAATACTAC |  |  |
| Total bacteria,  16S rRNA | (**F**) 16S | GATACATAGCCGACCTGAG | 98 | Periasamy, et al 2009* |
|  | (**R**) 16S | TCCATTGCCGAAGATTCC |  |  |
| *Enterococcus*, genus-level | (**F**) ENT1 (Fw) | TACTGACAAACCATTCATGATG | 112 | Ke, et al 1999 |
|  | (**R**) ENT2 (Rv) | AACTTCGTCACCAACGCGAAC |  |  |
| Total fungi,  5.8S-28S region | (**F**) FP 5.8S | GTGAATCATCGARTCTTTGAAC | 242-269 | Khot, et al 2009 |
|  | (**R**) RP 28S-1 | TATGCTTAAGTTCAGCGGGTA |  |  |
| PCR conditions: Thermal cycler (BioRad). The total reaction mixture was 20 μL per sample: 10 μL iTaq Universal SYBR® Green Supermix (Bio-Rad cat. #1725121), 1 μL of each 10 μM Forward and Reverse primers, 3 μL DNase-Free Water, and 5 μL of DNA template. All amplifications were followed by a Melt curve step, starting at 52 °C to 95 °C, in steps of 0.5 °C / 5 s. | | | | |
